# Plasma free DNA: Evaluation of temperature-associated storage effects observed for Roche Cell-Free DNA collection tubes

**DOI:** 10.11613/BM.2019.010904

**Published:** 2019-02-15

**Authors:** Dietmar Enko, Gabriele Halwachs-Baumann, Gernot Kriegshäuser

**Affiliations:** 1 Institute of Clinical Chemistry and Laboratory Medicine, General Hospital Steyr, Steyr, Austria; 2 Clinical Institute of Medical and Laboratory Diagnostics, Medical University Graz, Graz, Austria

**Keywords:** cell-free nucleic acids, pre-analytical phase, quality improvement

## Abstract

**Introduction:**

Standardized pre-analytical blood sample procedures for the analysis of circulating cell-free DNA (ccfDNA) are still not available. Therefore, the present study aimed at evaluating the impact of storage conditions related to different times (24 and 48 h) and temperatures (room temperature (RT) and 4 - 8 °C) on the plasma ccfDNA concentration of blood samples drawn into Cell-Free DNA collection tubes (Roche Diagnostics GmbH, Mannheim, Germany).

**Materials and methods:**

Venous blood from 30 healthy individuals was collected into five 8.5 mL Cell-Free DNA Collection Tubes (Roche Diagnostics GmbH) each. Plasma samples were processed at time point of blood collection (tube 1), and after storage under the following conditions: 24 h at RT (tube 2) or 4-8 °C (tube 3), and 48 h at RT (tube 4) or 4 - 8 °C (tube 5). Circulating cell-free DNA concentrations were determined by EvaGreen chemistry-based droplet digital PCR (ddPCR).

**Results:**

No statistically significant differences between median (interquartile range) plasma ccfDNA concentrations (ng/mL) at time point of blood collection (3.17 (2.13 – 3.76)) and after storage for 24 h (RT: 3.02 (2.41 – 3.68); 4-8 °C: 3.21 (2.19 – 3.46)) and 48 h (RT: 3.13 (2.10 – 3.76); 4-8 °C: 3.09 (2.19 – 3.50)) were observed (P values from 0.102 – 0.975).

**Conclusions:**

No unwanted release of genomic DNA from white blood cells could be detected in plasma samples after tube storage for 24 and 48 h regardless of storage temperature.

## Introduction

Molecular analysis of circulating cell-free deoxyribonucleic acid (ccfDNA) in human plasma is a promising diagnostic tool for a variety of clinical conditions. The discovery, that ccfDNA can also occur as a result of tumour cell turnover in the blood stream, has attracted wide interest. Molecular disease monitoring, including early detection of recurrence, prognostic assessment, and prediction of treatment response is increasingly used in clinics (*1*).

In daily clinical practice, the ccfDNA concentration in human plasma was shown to be affected by pre-analytical variables such as blood sampling, handling procedure variations and storage conditions (*2*). In this respect, optimal handling of blood samples between venepuncture and plasma preparation may be considered essential for downstream processes, as time delay and/or agitation could trigger the release of genomic deoxyribonucleic acid (DNA) from white blood cells (WBC) (*2*, *3*). One way to solve this problem is to perform ccfDNA extraction immediately after venepuncture (*2*, *4*).

Alternatively, cell-membrane stabilizing additives (*e.g.*, formaldehyde, glutaraldehyde), may be used to maintain the cells’ structural integrity (*4*). To address this issue, several manufacturers (*e.g.*, Roche Diagnostics GmbH, Mannheim, Germany; PreAnalytiX GmbH, Hombrechtikon, Switzerland; Streck, Omahoma, USA) have developed commercial blood collection tubes containing proprietary compounds, which impede WBC lysis.

The lack of standardization for sample processing procedures and storage conditions, which can affect the quantitative output of ccfDNA downstream diagnostic methods, requires laboratories to establish their own optimized pre-analytical phase. The current version of the manufacturer’s instruction does not contain information on the refrigerated transport/storage of blood drawn into Cell-Free DNA collection tubes (Roche Diagnostics GmbH). Therefore, the present study was conducted to evaluate the impact of different storage times (24 and 48 h) and temperatures (room temperature (RT) and 4 - 8 °C) on the plasma concentration of ccfDNA as determined by EvaGreen chemistry-based droplet digital polymerase chain reaction (ddPCR).

## Materials and methods

### Subjects

This observational study was conducted at the Institute of Clinical Chemistry and Laboratory Medicine of the General Hospital Steyr. The study period was from January to August 2018. A total of 30 healthy volunteers (24 females and 6 males with a median (range) age of 35 (20 – 55) years, which were recruited at our outpatient clinic in the morning between 8.00 and 10.00 a.m., were included. Ethical approval was obtained from the local ethics committee of Upper Austria (Linz, Austria). All participants provided written informed consent and the study was carried out according to the latest version of the Declaration of Helsinki.

### Blood sampling

Blood sampling was performed in the morning without obligatory fasting state. From all 30 study participants venous blood was collected into 5 evacuated 8.5 mL Cell-Free DNA collection tubes (Roche Diagnostics GmbH) each. To avoid haemolysis, the tourniquet was removed while filling the first tube, and all specimens were mixed by gently inverting the tubes, 8 to 10 times according to the manufacturer’s instructions.

### CcfDNA extraction

Immediately after blood collection, tube 1 was centrifuged at 1600xg for 15 minutes at RT, using a ROTOFIX 32 centrifuge (Hettich, Tuttlingen, Germany). Before centrifugation of the remaining tubes, conditions being equal, specimens were stored under each of the following conditions: 24 h at RT (tube 2) or 4 - 8 °C (tube 3), and 48 h at RT (tube 4) or 4 - 8 °C (tube 5). Following centrifugation, ccfDNA was extracted from 2 mL of plasma using the QIAamp Circulating Nucleic Acid Kit (PreAnalytiX GmbH) on the QIAsymphony instrument (Qiagen, Hilden, Germany) according to the manufacturer´s instruction. Circulating cell-free DNA was then eluted in a final volume of 60 µL and stored at - 70 °C until analysis.

### Droplet digital PCR

Circulating cell-free DNA was quantified by EvaGreen chemistry-based ddPCR using primers to generate a 110bp amplicon from the β-globin gene as described previously (*5*). Polymerase chain reaction amplification was performed in a 20 µL mixture containing 5 µL DNA template, each of the 2 β-globin-specific primers, 5´-ACACAACTGTGTTCACTAGC-3` (forward) and 5`-CAACTTCATCCACGTTCACC-3` (reverse), at 0.15 µmol/L, and 10 µL 2xEvaGreen ddPCR Supermix (Bio-Rad Laboratories). The reaction mixtures were transferred into each well of the droplet generator DG8 cartridge (Bio-Rad Laboratories, Hercules, CA) followed by the addition of 70 µL of droplet generation oil for EvaGreen (Bio-Rad Laboratories). After the generation of droplets using the QX200^TM^ droplet generator (Bio-Rad Laboratories), the entire droplet emulsion volume (40 µL) was loaded onto a ddPCR^TM^ 96-well plate (Bio-Rad Laboratories). Amplification was performed using a C1000 Touch^TM^ Thermal cycler (Bio-Rad Laboratories) and the following PCR conditions: 95 °C for 5 min and 40 cycles of 30 s at 95 °C and 1 min at 60 °C. After a final signal stabilization cycle at 4 and 90 °C for 5 min each, droplets were analysed using the QX200^TM^ Droplet reader (Bio-Rad Laboratories) together with the Bio-Rad QuantaSoft software (version 1.7.4.0917). All samples were analysed in duplicate and non-template controls were used to monitor for contamination and primer-dimer formation.

### Statistical analysis

Data distribution was calculated with the Kolmogorov-Smirnov test. Not normally distributed data were described in medians (interquartile ranges (Q1 – Q3)). The non-parametric Wilcoxon test was used for comparisons of ccfDNA concentrations between different storage conditions of blood samples. A P-value < 0.05 was considered statistically significant. Analyse-it^®^ software version 3.80 (Analyse-it Software, Ltd., Leeds, UK) was used for statistical analysis.

## Results

No statistically significant differences between median (Q1 – Q3) plasma ccfDNA concentrations (ng/mL) at time point of blood collection (3.17 (2.13 – 3.76)) and after storage for 24 h (RT: 3.02 (2.41 – 3.68); 4 - 8 °C: 3.21 (2.19 – 3.46)) and 48 h (RT: 3.13 (2.10 – 3.76); 4 - 8 °C: 3.09 (2.19 – 3.50)) were observed (P-values from 0.102 – 0.975) (Table 1). P-values comparing test tubes 2 *vs.* 3 and 4 *vs.* 5 were 0.440 and 0.504, respectively.

**Table 1:** Plasma ccfDNA concentrations detected in 30 individuals at time point of blood collection (t = 0), after storage of 24 and 48 h at RT and 4 - 8 °C

**N = 30**	**Storage**	**ccfDNA (ng/mL)**	**Difference (%)**	**P**
Tube 1	t = 0	3.17 (2.13 – 3.76)	/	/
Tube 2	24 h / RT	3.02 (2.41 – 3.68)	- 4.73	0.975
Tube 3	24 h / 4-8°C	3.21 (2.19 – 3.46)	+ 1.26	0.387
Tube 4	48 h / RT	3.13 (2.10 – 3.76)	- 1.26	0.102
Tube 5	48 h / 4-8°C	3.09 (2.19 – 3.50)	- 2.52	0.465
ccfDNA – circulating cell-free DNA. t = 0 – time point of blood sample collection. h – hours. RT – room temperature. Differences of ccfDNA concentrations observed for various storage conditions were evaluated using the Wilcoxon test. Data are presented as median (Q1 – Q3). P < 0.05 was considered statistically significant.				

## Discussion

No unwanted release of genomic DNA from WBC could be detected in plasma samples obtained from blood drawn into Cell-Free DNA collection tubes (Roche Diagnostics GmbH) after tube storage for 24 and 48 h at RT and 4 - 8 °C.

In comparison, a recently published study by Zhao *et al.*, investigating 6 healthy volunteers, reported marginal genomic DNA release from leukocyte lysis in Roche Cell-Free DNA collection tubes after a 7-day storage at RT (*4*). Similar results were shown by Nikolaev *et al.*, who could not detect significant genomic DNA contamination by capillary electrophoresis in the same type of tube kept at RT for 1 week. However, the authors of the latter study did not provide the number of probands included in their work (*6*).

It has been demonstrated that *in vitro* haemolysis may associate with increased plasma ccfDNA concentrations (*2*). Here, visible hemolysis was present in all 30 sample tubes stored for 48 h at 4 - 8 °C, however, this fact was not accompanied by the release of genomic DNA from WBC. This observation adds value to the pre-analytical performance of Cell-Free DNA collection tubes (Roche Diagnostics GmbH), especially considering the fact that, current literature lacks data on the stability of WBC in blood samples stored and shipped at 4 - 8 °C.

In routine diagnostics, the use of suitable blood collection tubes for liquid profiling procedures has become highly important (*7*, *8*). Various types of sample tubes for blood drawing and storage have demonstrated to significantly influence ccfDNA yield and quality (*9*). For example, preservative-containing tubes were shown to prevent the contamination of ccfDNA with genomic DNA in blood samples with prolonged transfer or storage times (*9*, *10*). However, defining optimal temperatures for sample shipment and storage remains one of the main pre-analytical issues. Our findings suggest that, in order to avoid artificial *in vitro* haemolysis, blood samples contained in Cell-Free DNA collection tubes (Roche Diagnostics GmbH) should neither be transported nor stored refrigerated. The standardization of pre-analytical procedures for ccfDNA analysis in human plasma is a great challenge in the next few years. In order to become part of daily routine and with respect to the appropriate clinical interpretation of liquid profiling results, further studies are needed to fully define and optimise blood collection and storage conditions for subsequent ccfDNA measurements.

This study is limited by the fact, that neither the haemolysis index nor haemolysis parameters (*e.g.*, potassium, lactate dehydrogenase, haptoglobin, bilirubin, concentration of free haemoglobin) were measured.

In conclusion, no unwanted release of genomic DNA from WBC could be detected in plasma samples obtained from blood drawn into Cell-Free DNA collection tubes (Roche Diagnostics GmbH) after tube storage for 24 and 48 h regardless of storage temperature.
